# Phagocytosis and Efferocytosis by Resident Macrophages in the Mouse Pancreas

**DOI:** 10.3389/fendo.2021.606175

**Published:** 2021-05-25

**Authors:** Kristel Parv, Nestori Westerlund, Kevin Merchant, Milad Komijani, Robin S. Lindsay, Gustaf Christoffersson

**Affiliations:** ^1^ Department of Medical Cell Biology, Uppsala University, Uppsala, Sweden; ^2^ Science for Life Laboratory, Uppsala University, Uppsala, Sweden

**Keywords:** pancreas, macrophages, phagocytosis, efferocytosis, *in vivo* imaging, type 1 diabetes

## Abstract

The tissue microenvironment in the mouse pancreas has been shown to promote very different polarizations of resident macrophages with islet-resident macrophages displaying an inflammatory “M1” profile and macrophages in the exocrine tissue mostly displaying an alternatively activated “M2” profile. The impact of this polarization on tissue homeostasis and diabetes development is unclear. In this study, the ability of pancreas-resident macrophages to phagocyte bacterial and endogenous debris was investigated. Mouse endocrine and exocrine tissues were separated, and tissue-resident macrophages were isolated by magnetic immunolabeling. Isolated macrophages were subjected to flow cytometry for polarization markers and qPCR for phagocytosis-related genes. Functional *in vitro* investigations included phagocytosis and efferocytosis assays using pH-sensitive fluorescent bacterial particles and dead fluorescent neutrophils, respectively. Intravital confocal imaging of *in situ* phagocytosis and efferocytosis in the pancreas was used to confirm findings *in vivo*. Gene expression analysis revealed no significant overall difference in expression of most phagocytosis-related genes in islet-resident vs. exocrine-resident macrophages included in the analysis. In this study, pancreas-resident macrophages were shown to differ in their ability to phagocyte bacterial and endogenous debris depending on their microenvironment. This difference in abilities may be one of the factors polarizing islet-resident macrophages to an inflammatory state since phagocytosis has been found to imprint macrophage heterogeneity. It remains unclear if this difference has any implications in the development of islet dysfunction or autoimmunity.

## Introduction

Type 1 diabetes is a disease with autoimmune features where insulin producing beta cells are progressively lost leading to hyperglycemia and dependence on exogenous insulin. Hallmark histopathological features include the accumulation of lymphocytes and macrophages in and around the pancreatic islets (1). The cells from the adaptive immune system have been extensively studied in this disease whereas the specific contributions and functions of innate immune cells, including macrophages, during disease onset have gathered less attention.

In work by the Unanue group, a surprising dichotomy in the phenotypes of pancreatic macrophages in mice has been uncovered (2, 3). Macrophages residing in the islets of Langerhans (endocrine-resident) are classically activated and exhibit an inflammatory phenotype (also called “M1 macrophages”) whereas macrophages residing in the exocrine pancreas are mostly alternatively activated, and exhibit an anti-inflammatory phenotype (also called “M2 macrophages”) (4). The complete functional impact of the very specific polarization of pancreatic macrophages in the mouse is however not fully clear.

Endocrine-resident macrophages have been shown to be in close contact with both islet capillaries and beta-cells and can take up insulin granule contents and present these on MHC-II (5). Supporting their active role in controlling the inflammatory environment, endocrine-resident macrophages have also been observed to act as “gatekeepers” for the entry of lymphocytes into islets during onset of T1D in mouse models (6). Further observations in the *op/op* mouse (inactivating mutation in the macrophage colony stimulating factor-1 gene (*Csf1*) resulting in the absence of CSF-1) lends data to suggest that the endocrine-resident macrophage also likely has homeostatic properties since lacking macrophages in islets leads to poorly developed islets (7).

An important homeostatic function of macrophages is clearance of apoptotic cells and debris from the normal turnover of cells in the body (8). A comprehensive study on efferocytosis (clearance of endogenous cells) by macrophages in different organs of mice found expression level of the mannose receptor (Mrc-1/CD206/MMR), and a general M2-phenotype, to be the main factors correlating to the efferocytic capacity of tissue-resident macrophages (9). Since endocrine-resident macrophages in mice lack the expression of MMR (2), this poses the question whether these macrophages are less prone to phagocytosis and efferocytosis, possibly resulting in aggregation of cellular debris in events of intra-islet apoptosis and in clearance of invading bacteria.

We decided to investigate the function of pancreas-resident macrophages in healthy mice with regards to their efficiency to take up bacteria by phagocytosis and apoptotic endogenous cells by efferocytosis. We found no difference in the expression of phagocytosis-related genes between endocrine-resident and exocrine-resident macrophages. Neither were endocrine-resident macrophages impaired in their ability to engulf bacteria or apoptotic cells *in vitro* or *in vivo*, showing that despite the inflammatory phenotype of endocrine-resident macrophages, they still likely contribute to homeostatic processes.

## Materials and Methods

### Animals

Male C57Bl/6 (wild-type) mice were purchased from Taconic, C57Bl/6*^Ly6g^* ^(tm2621(Cre-tdTomato)Arte^ (also named ‘Catchup’) mice (10) bred in-house were used as a source of red-fluorescent neutrophils, and CX_3_CR1*^+/Gfp^* (B6.129P2(Cg)-*Cx3cr1^tm1Litt^*/J) mice (11) bred in-house were used for imaging of pancreatic macrophages. Mice used in experiments weighed 25-30 g. Mice had free access to tap water and pelleted food throughout the study. All experiments were approved by the Uppsala Region Laboratory Animal Ethics Board (number 5.9.18-03603/2018).

### Tissue Staining and Human Histopathology

Mouse pancreata fixed in 4% paraformaldehyde were snap-frozen and sectioned into 20 µm sections. The tissues were then stained for MMR and F4/80 using antibodies listed in **Supplemental Table 1** and imaged using a Leica SP8 scanning confocal microscope.

Human biobanked normal pancreas tissue excised from three patients undergoing surgery for pancreatic neoplasms had been snap-frozen, and sections were fixed in 4% paraformaldehyde before being stained for MMR and CD68 using antibodies listed in **Supplemental Table 1** and imaged using a Leica SP8 scanning confocal microscope. Ethical approval for use of the biobanked material was provided by the Regional Ethics Review Board.

### Neutrophil Isolation and Apoptosis Induction

Mouse bone marrow- (BM) and spleen-derived neutrophils were isolated from heterozygous Catchup mice using a positive selection kit for Ly6G^+^ cells (Miltenyi Biotec, #130-092-332). Isolated neutrophils were cultured for 24 h at 37°C/5% CO_2_ in RPMI-1640 media with 2% FBS to induce apoptosis. Neutrophil apoptosis was determined using the Annexin V Apoptosis Kit with 7-AAD (Biolegend; #640922) according to the manufacturer’s instructions.

### Pancreatic Macrophage Isolation

Endocrine and exocrine fractions of the pancreas were separated using a density gradient approach as described earlier (12). Single cell suspensions of the endocrine and exocrine fractions were prepared by incubating the fractions for 5 min at 37°C in 3 ml of non-enzymatic cell dissociation buffer (Thermo Fisher Scientific; #13151014), followed by rapidly pipetting the solution and further incubation for 3 min at 37°C. Thereafter 10 ml of buffer (RPMI-1640, 2% FBS, 2 mM EDTA) was added and suspensions were filtered through a 70 µm cell strainer. Following centrifugation at 300xg for 10 min, anti-F4/80 microbeads (Miltenyi Biotec; #130-110-443) were used for positive selection of macrophages from endocrine and exocrine fractions according to the manufacturer’s instructions. The fractions were run twice through the magnetic columns to reach a purity of 90% for endocrine tissue and 55% for exocrine tissue, see **Supplemental Figure 1** for representative flow cytometry plots.

### Gene Expression Analysis

Isolated fractions of exocrine and endocrine macrophages pooled from two pancreata per observation were subjected to mRNA isolation using a commercial kit (Single Cell RNA Purification Kit, Norgen Biotek). Synthesis to cDNA was done using the High Capacity cDNA Synthesis kit (ThermoFisher). Custom oligos were from ThermoFisher (primer sequences in **Supplemental Table 2**). Gene amplification was detected using Fast Sybr Green (ThermoFisher) and read on a QuantStudio 5 qPCR machine (ThermoFisher).

### Pancreatic Macrophage Characterization

Exocrine and endocrine macrophages were stained for various M1 and M2 macrophage markers. Briefly, after separation of the exocrine and endocrine fraction as described above, cells were stained with Violet Dead Cell Stain Kit (Invitrogen; #L34955). Thereafter cells were incubated with BD Mouse Fc Block (BD Biosciences; #553141) in RPMI-1640 containing 10% FBS for 10 minutes at room temperature, followed by adding optimal concentrations of extracellular antigen primary antibodies (**Supplemental Table 1**) for 15 minutes on ice. Cells were washed twice with buffer (RPMI-1640, 2% FBS, 2 mM EDTA), followed by secondary antibody staining (if applicable) for 15 min on ice. Thereafter cells were fixed by adding Intracellular Fixation buffer (eBioscience; #88-8824-00) for 15 min at room temperature and washed twice with Permeabilization Buffer (eBioscience; #88-8824-00). The cells were then stained with intracellular antigen antibodies (**Supplemental Table 1**) in Permeabilization buffer for 30 min on ice. The cells were then washed in Permeabilization buffer twice and resuspended in Intracellular Fixation buffer. Cells were analyzed using CytoFlex (Beckman Coulter) or Northern Lights 2000 (Cytek) flow cytometers and data was processed using FlowJo software (BD).

### Phagocytosis and Efferocytosis Assays

For determining phagocytic capacity of macrophages, pHRodo Red *E.coli* BioParticles Conjugate for Phagocytosis kit (ThermoFisher) was used at a concentration of 1 mg/mL according to the manufacturer’s instructions. The pHRodo Red conjugate is non-fluorescent outside the cells but will be brightly fluorescent in the low pH environment of phagosomes. Briefly, isolated macrophages pooled from the exocrine and endocrine fractions of four pancreata were cultured for 30 min with pHRodo *E.coli* BioParticles at 37°C. For determining efferocytic capacity of macrophages, isolated macrophages were cultured for 30 min with tdTomato-fluorescent apoptotic neutrophils (from Catchup mice) at 37°C in a 1:30 ratio (each tube contained approximately 2,000 macrophages and 60,000 neutrophils). These experiments were performed in 1 mL complete culture medium (RPMI1640 supplemented with 10% FCS and penicillin/streptomycin). Following phagocytosis and efferocytosis assays, cell suspensions were stained with antibodies against CD45 and F4/80 (**Supplemental Table 1**). Cells were analyzed using a CytoFlex flow cytometer with CytExpert software (Beckman Coulter).

### Cytokine Analysis

Supernatants were collected from the phagocytosis and efferocytosis experiments conducted during 4 h instead of 30 min, and diluted 1:2 in Diluent 41 (Mesoscale Diagnostics). Diluted supernatants were added in triplicates on a Vplex Proinflammatory Panel 1 multiplex protein assay (Mesoscale Diagnostics) and read on a Sector S 600 instrument (Mesoscale Diagnostics).

### *In Vivo* Confocal Imaging

CX_3_CR1*^+/Gfp^* mice were injected intravenously with either pHrodo *E.coli* bioparticles (50 µl of the supplied stock solution) or 10^7^ Catchup tdTomato^+^ neutrophils. After 4 and 24 h, respectively, mice were anesthetized by spontaneous inhalation of isoflurane (Abbvie) and injected intravenously with anti-CD31 (390) antibody tagged with Alexa Fluor 647 (**Supplemental Table 1**). The pancreas was exposed and mounted for intravital imaging with a suction ring as previously described (13, 14). The 3D printed suction ring and imaging setup has been previously described by us (15). Images were acquired using a confocal laser scanning microscope (Leica TCS SP8).

### Image Analysis

Analyses for all images were performed using Imaris (Bitplane) and FIJI/ImageJ (NIH). More specifically, for quantifying uptake of red-fluorescent pHrodo *E.coli* bioparticles or Catchup neutrophils, green-fluorescent macrophages in confocal z-stacks were transformed into volume surfaces and masked for red fluorescence. This allows for distinction of only the red-fluorescent particles present within the macrophages. To distinguish intra-islet macrophages from exocrine-resident macrophages, the reflection signal from the islet was transformed into a volume surface and masked for green fluorescence. This allows for distinction of intra-islet-resident macrophages. The total number of macrophages and macrophages with intracellular red fluorescence were counted manually.

For tissue sections stained for F4/80 (mouse) or CD68 (human) and CD206, cells staining positive for each antibody were counted using the spots-function in Imaris. Since a small fraction of the CD206 signal does not label macrophages, only double-positive cells were counted as CD206-positive. The area of islets and exocrine tissue was measured using ImageJ.

### Validation of Results Using Tabula Muris Senis

Tabula Muris Senis (16) pancreatic FACS dataset processed files from figshare (https://figshare.com/articles/dataset/Tabula_Muris_Senis_Data_Objects/12654728) were utilized for this study. Only cell types identified as leukocytes in (16) were analyzed. Analysis of scRNA-seq data sets were performed with Scanpy Package (v1.6.0) (17) in Python Package (v3.6.10), with counts log-normalised using size factor normalization. Macrophages were defined with *Emr1* expression > 0.5 (log-normalised counts).

### Statistics

Results are presented as mean ± standard deviation. Type of statistical test for each analysis is mentioned in the figure legends. *P*-values of less than 0.05 were considered statistically significant.

## Results

### Exocrine and Endocrine Pancreatic Macrophage Profile Under Steady State

**Figure 1:**
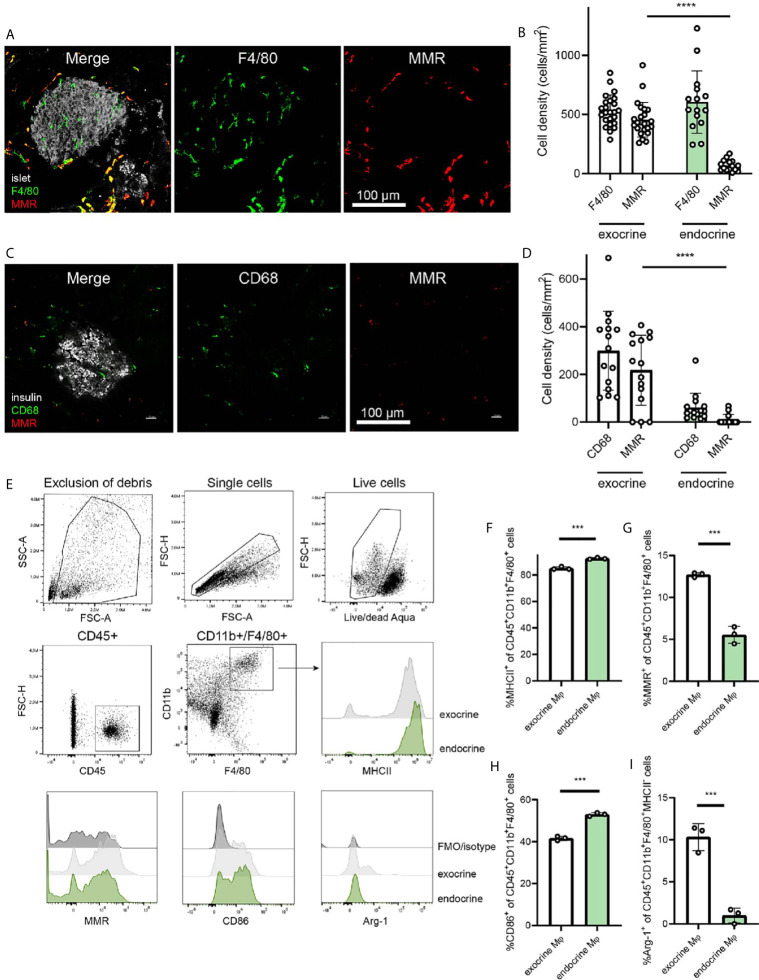
Endocrine-resident macrophages are skewed to an inflammatory phenotype. **(A)** Immunofluorescence of the mouse pancreas reveals total absence of the mannose receptor (MMR) in endocrine-resident macrophages, whereas exocrine-resident macrophages express this receptor to a large extent (islets in grey, laser reflection, pan-macrophage marker F4/80 in green, MMR in red). **(B)** Quantification of immunofluorescence stainings for the pan-macrophage marker F4/80 and MMR in the pancreas showed that more than 90% of macrophages in the exocrine tissue expressed MMR and only a few percent of the endocrine-resident macrophages did (n=9 mice, 15 islets, 24 exocrine regions, ****P < 0.0001, Mann-Whitney U test). **(C)** Representative image of staining human pancreata for CD68 (green), MMR (red), and insulin (grey) **(D)** The expression of MMR in macrophages in human endocrine tissue was found to be lower than for macrophages in the exocrine tissue (n=3 donors, ****P < 0.0001, Mann-Whitney U test). **(E)** Gating strategy for endocrine- and exocrine-resident macrophages from mouse pancreas, including representative flow histograms of MHCII, MMR, CD86, and Arg-1 expression (representative of three independent experiments). **(F)** Endocrine-resident macrophages expressed significantly more MHCII, **(G)** significantly less MMR, **(H)** significantly more CD86, and **(I)** significantly less Arg-1 (of MHC-II^-^ populations) than exocrine-resident macrophages (***P < 0.001, Mann-Whitney U test).

The differing phenotypes of macrophages in the pancreas have been characterized previously (2), and the dichotomy between endocrine-resident and exocrine-resident macrophages becomes very evident when staining for the mannose receptor 1 (Mrc1/MMR/CD206) (**Figure 1A**). Macrophages within mouse islets do not express MMR, whereas macrophages in the exocrine tissue are largely positive for this receptor (**Figure 1B**).

For assessing the expression of MMR in the human pancreas, we stained pancreata from three human non-diabetic donors. Similar to the mouse pancreas, macrophages in the endocrine tissue expressed less MMR than macrophages in the exocrine tissue (**Figure 1C**). Macrophages in exocrine tissue were however expressing MMR to a higher extent, suggesting a similar dichotomy also in human pancreata (**Figure 1D**).

Macrophages (CD45^+^CD11b^+^F4/80^+^) of the exocrine and endocrine mouse pancreas were further characterized for their phenotypic polarization by flow cytometry (**Figure 1E**). Endocrine-resident macrophages had higher expression of MHCII compared to exocrine-resident macrophages (**Figure 1F**; mean fluorescence intensity (MFI) 5.7 ± 0.53*10^6^ *vs.* 2.4 ± 0.17*10^6^, respectively, *P*<0.0001) as previously found (2). Also by flow cytometry, endocrine-resident macrophages expressed less MMR than exocrine-resident macrophages (**Figure 1G**; MFI 3.2 ± 0.48*10^4^ *vs.* 8.9 ± 0.20*10^4^, respectively, *P*<0.0001). Further, endocrine-resident macrophages expressed more CD86 (**Figure 1H**; MFI 9.0 ± 0.23*10^3^ *vs.* 7.6 ± 0.10*10^3^, respectively, *P*=0.0008), and less Arginase-1 by percent MHCII^-^ macrophages (**Figure 1I**; MFI 1.0 ± 0.020*10^3^ *vs.* 8.0 ± 1.6*10^3^, respectively, *P*=0.1) than exocrine-resident macrophages, recapitulating the previously reported skewing of endocrine-resident macrophages to an inflammatory phenotype.

### Expression of Phagocytosis-Related Genes

**Figure 2:**
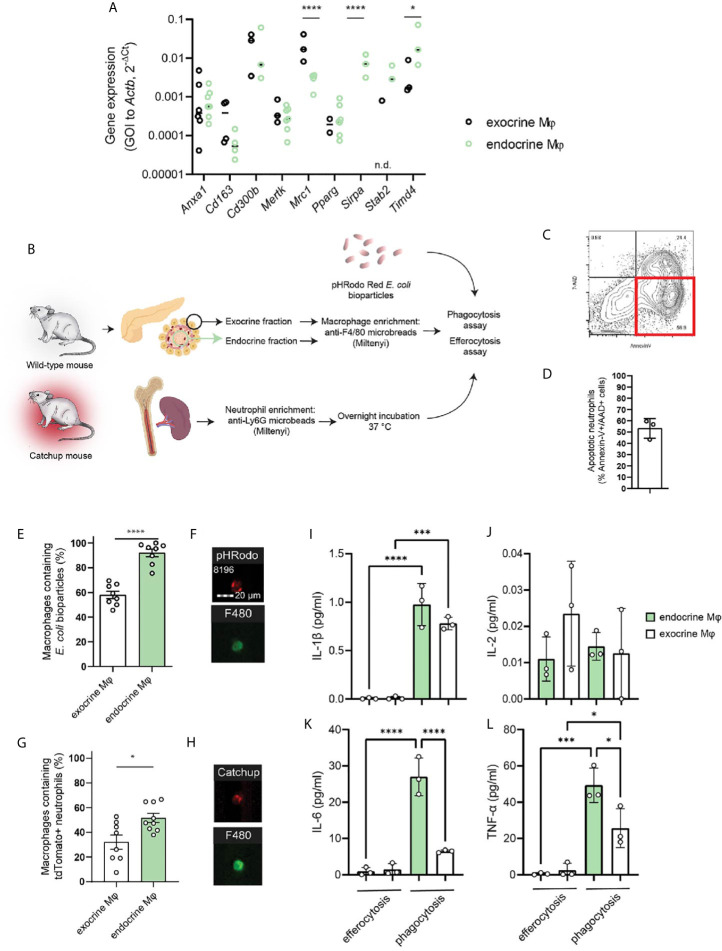
Endocrine-resident macrophages are efficient in phagocytosis and efferocytosis *in vitro*. **(A)** A selection of phagocytosis-related genes were assessed by qPCR in macrophages from the endocrine and exocrine fractions, respectively. No distinct pattern suggesting functional impairment between macrophages from the two compartments could be observed other than the very low expression of MMR in endocrine-resident macrophages (n=3-7 mice per group, *P > 0.05, ****P ≤ 0.0001, 2-way ANOVA, n.d.=not detected). **(B)** Schematic of workflow for phagocytosis and efferocytosis assays. **(C, D)** Flow plot and quantification of the fraction of Catchup neutrophils being in a pre-apoptotic state for the efferocytosis assay. **(E)** Endocrine-resident macrophages were significantly more efficient in phagocytosis of pHrodo *E.coli* bioparticles than exocrine-resident macrophages (n=8 mice, ****P < 0.0001, Mann-Whitney U test). **(F)** Images from the FlowSight flow cytometer demonstrating the internalization of red-fluorescent bacterial particles and positivity for the pan-macrophage marker F4/80. **(G)** Endocrine-resident macrophages were also more efficient in efferocytosis of dying neutrophils than exocrine-resident macrophages (n=9 mice, *P < 0.05, Mann-Whitney U-test). **(H)** Images from the FlowSight flow cytometer demonstrating the internalization of tdTomato-red fluorescent neutrophil particles and positivity for the pan-macrophage marker F4/80. **(I–L)** Cytokine content in supernatants from efferocytosis and phagocytosis experiments for endocrine- and exocrine-resident macrophages (n=3 separate experiments in which macrophages from 2 mice were pooled per datapoint, *P < 0.05, ***P < 0.001, ****P < 0.0001, one-way ANOVA).

We isolated macrophages from the endocrine and exocrine compartments of the pancreas by first separating the islets from the exocrine fraction through enzyme digestion and density-gradient centrifugation, dissociating the tissues, and isolating resident macrophages using anti-F4/80 magnetic beads. A selection of phagocytosis-related genes was then assessed by RT-qPCR in the two different fractions. As expected, there was a great difference in expression of *Mrc1* (MMR) with endocrine-resident macrophages expressing significantly less of this gene. Also *Timd4*, which encodes Tim-4 – a receptor involved in engulfment of apoptotic cells, was significantly more expressed in endocrine-resident macrophages. None of the other assessed phagocytosis/efferocytosis-related genes (*Anxa1, Cd163, Cd300b, Mertk, Pparg, Sirpa, Stab2*) were differentially expressed through the two fractions of macrophages (**Figure 2A**). We could thus not discern any obvious skew in any of the subsets for having extraordinary phagocytic abilities on the mRNA level. Further, we validated our findings with data on exocrine and endocrine leukocytes from the open single-cell sequencing database *Tabula Muris Senis* (16). Also in this data, apart from significant differences in *Mrc1* and *Cd163* expression, no apparent pattern in the differences between these genes could be found (**Supplemental Figure 2**).

### Endocrine Macrophages Phagocytose More Than Exocrine Macrophages

To test whether lack of MMR expression on endocrine macrophages predicts lower phagocytic capacity compared to exocrine macrophages, we purified exocrine- and endocrine-resident macrophages for phagocytosis and efferocytosis assays using the method described above. Isolated macrophages were incubated with *E.coli* particles loaded with the pH-sensitive probe pHrodo which will increase its fluorescence when present in the acidic endosomes following phagocytosis (**Figure 2B**). We found that whilst 58 ± 3% of the exocrine macrophages had taken up the bacterial particles, 92 ± 3% of the endocrine-resident macrophages had taken up the same (**Figures 2E, F**). For comparison, we performed the same assessment for cultured mouse bone marrow-derived macrophages (BMDMs) pushed into either M1 or M2 states. Cultured M2 BMDMs were in our hands expressing twice the amount of MMR of M1 BMDMs (MFI 2.23 ± 0.42*10^4^ *vs.* 1.11 ± 0.18*10^4^, respectively, *P*=0.01). We found that M1 macrophages were more efficient phagocytes than M2 macrophages (MFI 2.24 ± 0.25*10^5^ *vs.* 1.73 ± 0.15*10^5^, respectively, *P*=0.02).

### Endocrine-Resident Macrophages Are Efficient in Efferocytosis

The paper identifying the correlation between macrophage MMR expression and phagocytic capacity primarily investigated the engulfment of dead endogenous cells, efferocytosis (9). The most prevalent cell to be subject to efferocytosis is the short-lived neutrophil. To assess the efficiency of efferocytosis in pancreatic macrophages, we used neutrophils isolated from Catchup mice (red-fluorescent tdTomato expression in neutrophils driven by Ly6G expression). As neutrophils generally will not survive prolonged *in vitro* culture, these neutrophils were incubated overnight following isolation to induce apoptosis in the main fraction of the cells. Following overnight culture at 37°C/5% CO_2_, and 58.25 ± 1.35% of the neutrophils were found to be early apoptotic (AnnexinV^+^/7-AAD^-^, **Figures 2C, D**). The neutrophils were incubated with pancreatic macrophages, and in this assay, 32 ± 6% and 52 ± 4% of the exocrine- and endocrine-resident macrophages, respectively, were tdTomato-fluorescent, indicating an ongoing engulfment of an apoptotic neutrophil by a macrophage (**Figures 2G, H**). The ratios of macrophages to apoptotic neutrophils were not significantly different between the two groups (1:21 ± 11 in the exocrine fraction *vs.* 1:42 ± 15 in the endocrine fraction).

Whether the macrophages from the two different compartments responded in different ways to *E.coli* particles or dying neutrophils was assessed by measuring cytokine release in supernatants from the phagocytosis and efferocytosis assays after 4h of incubation. Using a multiplexed assay, we found that of the detectible cytokines, the phagocytic response to bacterial particles elicited a much greater release of IL-1β, IL-6, and TNF-α than the efferocytic response to dead cells (**Figures 2I–L**). During phagocytosis, secretion of these cytokines were also higher in macrophages from the endocrine compartment than the exocrine compartment, likely reflecting their initial proinflammatory phenotype. The secretion of IL-2 was similar across the different stimuli and compartments (**Figure 2J**).

### In Vivo Phagocytosis and Efferocytosis by Pancreatic Macrophages

**Figure 3:**
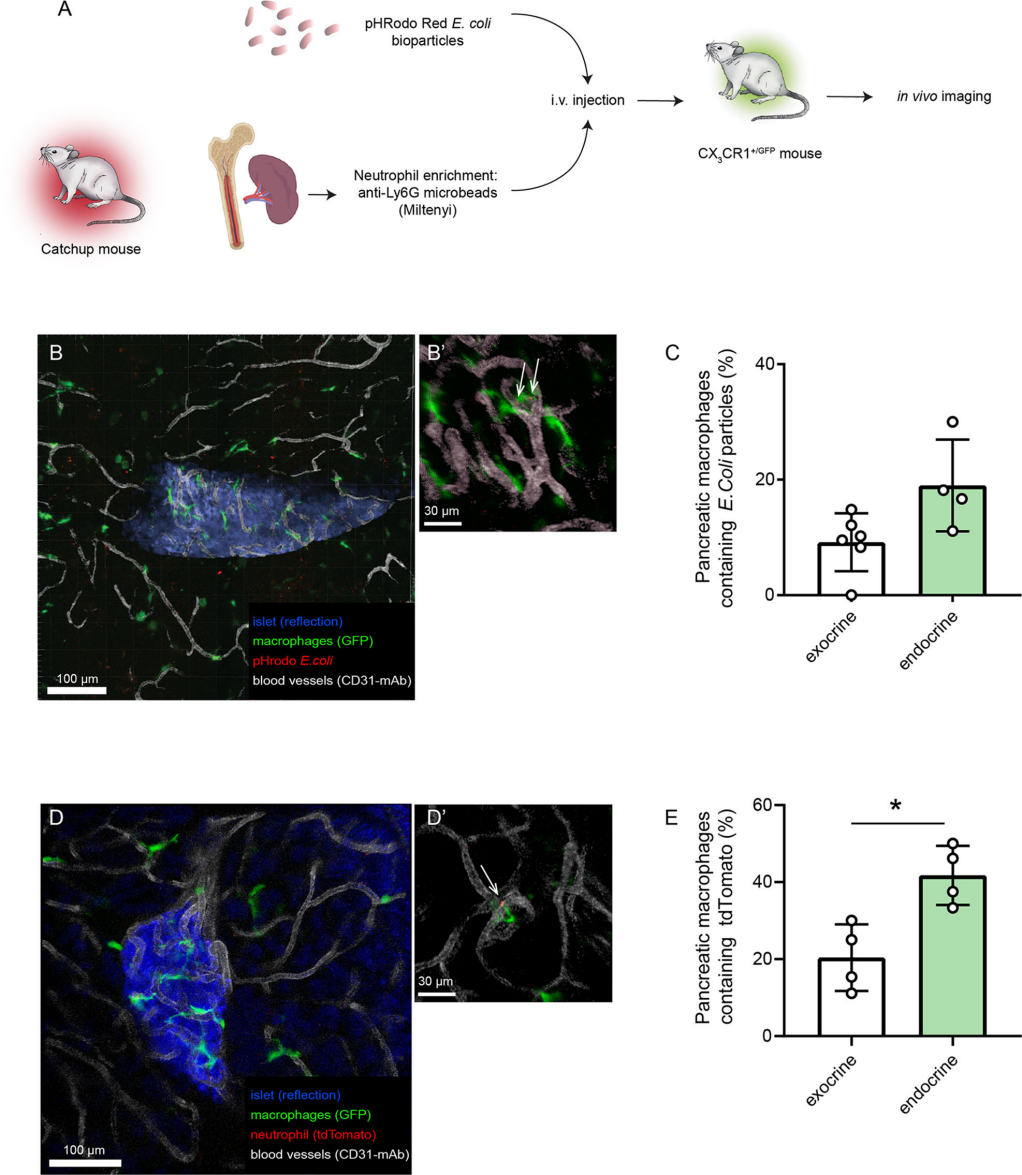
Endocrine-resident macrophages are efficient in phagocytosis and efferocytosis *in vivo*. **(A)** Schematic of workflow for *in vivo* assessment of phagocytosis and efferocytosis in pancreatic macrophages by intravital confocal imaging. **(B)** Confocal z-projection of an intravital recording showing an islet (blue), blood vessels (white), macrophages (green), and pHrodo *E.coli* bioparticles (red). **(B’)** Closeup showing pHrodo signals within macrophages (arrows). **(C)** Quantification of phagocytosis in pancreatic macrophages (n=4, Mann-Whitney U test). **(D)** Confocal z-projection of an intravital recording showing an islet (blue), blood vessels (white), macrophages (green), and tdTomato-red particles of engulfed neutrophils (red). **(D’)** Closeup showing tdTomato signal within a macrophage (arrow). **(E)** Quantification of efferocytosis in pancreatic macrophages (n=4, *P < 0.05, Mann-Whitney U test).

As the microenvironment in the pancreas likely is driving the polarization of the tissue-resident macrophages, *in vitro* assessment of their function may therefore introduce phenotype shifts. In order to have an *in vivo* comparison to our *in vitro* data on pancreatic macrophage phagocytosis, we evaluated the phagocytic and efferocytic capacities of pancreatic macrophages by intravital imaging. Either pHrodo *E.coli* bioparticles or neutrophils isolated from Catchup mice were intravenously injected into different groups of CX_3_CR1*^+/Gfp^* mice (green-fluorescent macrophages, **Figure 3A**). After 4 and 24 hours, respectively, intravital confocal imaging of the pancreas was performed, and macrophages which had engulfed bacterial particles or tdTomato^+^ neutrophils could be observed having an intracellular red-fluorescent signal (**Figures 3B, C**). Quantification of these data using image analysis masking functions showed that also *in vivo*, endocrine-resident macrophages showed a tendency (*P*=0.12) to follow the same pattern seen in the *in vitro* assays regarding phagocytosis with a slightly higher fraction of endocrine-resident macrophages containing engulfed bacterial particles (**Figure 3D**). For efferocytosis of dying neutrophils, endocrine-resident macrophages had significantly more intracellular tdTomato residues than macrophages residing in the exocrine tissue (**Figure 3E**). Even though total percentages were lower than in the *in vitro* assays, these results show that the level of expression of MMR does not predict the phagocytic capacity of subsets of pancreatic macrophages.

## Discussion

In reaching for a better understanding of the underlying mechanisms that congregate to the onset of T1D, knowing the functions of resident immune cells in the pancreas is crucial. Macrophages, a cell type with a multitude of homeostatic and pathophysiological functions, make up a major part of the resident immune cells in mouse islets of Langerhans (2), and is present also in human islets where the leukocyte composition is somewhat more diverse (18, 19). The microenvironment in mouse islets cause resident macrophages here to take on an inflammatory phenotype whereas macrophages in the surrounding exocrine tissue are mostly of an anti-inflammatory phenotype. Previous literature had identified inflammatory macrophages to be less efficient in clearing tissue debris (9), and we thus became interested in investigating some homeostatic functions of endocrine-resident macrophages. We found that despite lacking the MMR receptor – previously shown to be the primary marker correlating to efficient phagocytosis (9) – endocrine-resident macrophages were very efficient in phagocyting bacterial particles and clearing apoptotic cells by efferocytosis both *in vitro* and *in vivo*.

In their role as a resident, antigen presenting cell in the islets of Langerhans, macrophages have been associated with the initial steps in triggering an autoimmune attack against the beta-cells. Since the primary identified susceptibility loci for T1D are in the class II human leukocyte antigen (HLA) genes in the major histocompatibility complex (MHC-II) (20), the antigen presentation capability of these cells and their proximity to the beta cells have led to hypotheses regarding their ability to induce an antigen-directed response against e.g. released insulin granule contents (21). Endocrine-resident macrophages have also been found to act as regulators in limiting access to the islet parenchyma to T cells. By intravital imaging, T cell intravascular arrest and extravasation was observed in close proximity to endocrine-resident macrophages in a chemokine-dependent manner, and the depletion of macrophages limited this extravasation (6). Macrophage depletion has also been found to protect from the onset of T1D in the NOD and LCMV-RIP-GP mouse models (22, 23). Taken together, data from mouse models of T1D point to a pivotal role of endocrine-resident macrophages in the initial steps leading up to antigen-dependent T cell targeting of beta-cells.

The past few decades have seen great developments in the detailed understanding of macrophages in different organs and different situations; in homeostasis, in tissue restoration, and in pathophysiology. From the early discovery that macrophages are a population of polarized cells; classically activated/inflammatory/M1, and alternatively activated/anti-inflammatory/M2 (24), a wide range of subsets have been identified on that spectrum. Most tissue-resident macrophages display an M2 phenotype (25, 26), and thus the finding that endocrine-resident macrophages are distinctly polarized into an M1 phenotype, and very different from tissue-resident macrophages in the exocrine pancreas was very surprising (2). The functional relevance of this dichotomy is however still unclear. A recent paper has however provided detailed data on the development of the inflammatory environment in pancreatic islets of the NOD mouse through RNA sequencing of isolated immune cells exposing activation programs in the macrophage population through the disease course (27).

An intriguing finding from a study where the phagocytic ability of tissue-resident macrophages across a range of organs in the mouse body was that the level of expression of the mannose receptor (MMR) was the factor that correlated most positively to efferocytosis of apoptotic endogenous cells (9). In our own observation and those by others (2), we have found that endocrine-resident macrophages do not express MMR. This does also not seem to be the case for macrophages in the human pancreas as seen sections from three donated human pancreata, where the density of MMR^+^ macrophages was higher in exocrine tissue than in islets. This poses the question whether the endocrine-resident macrophages have a functional impairment in phagocytosis and efferocytosis, which could in turn have implications to islet homeostasis. Endocrine-resident macrophages have previously been found to take up beta cell fragments in non-diabetic, steady-state conditions, but were not compared to resident macrophages in other tissues (28).

We thus set out to investigate phagocytosis and efferocytosis in pancreatic macrophages. We found no major differences in the level of expression of a range of phagocytosis-related genes between exocrine (high expression of MMR) and endocrine (no expression of MMR) macrophages. In the functional assessment of their phagocytic abilities, we used commercially available *E.coli* particles which will turn brightly fluorescent once internalized in phagosomes (29). Following incubation of macrophages isolated from the mouse pancreas with bacterial particles, we used imaging flow cytometry as a readout to make sure that the signal from the *E.coli* had indeed been internalized in the macrophages and was not only adhering to the outer cell membrane. A similar procedure was performed using red-fluorescent apoptotic neutrophils for assessing efferocytosis. In both of these assays, endocrine-resident macrophages were more efficient in engulfing bacteria or dying cells. To the background of the paper stating MMR expression to correlate to phagocytic efficiency, this was somewhat surprising. We did however also find that cultured macrophages derived from mouse bone marrow and pushed into M1 or M2 states that the inflammatory M1 macrophages were more efficient in phagocytosis. The different activation states of endocrine- and exocrine-resident macrophages was reflected in their secretion of cytokines in response to bacterial particles. Endocrine-resident macrophages secreted significantly more IL-6 and TNF-α than exocrine-resident macrophages. Interestingly, the response to efferocytosis of dead cells induced a much lower secretion of cytokines than the phagocytic response to bacteria. Efferocytosis is a homeostatic process which is constantly ongoing physiologically, and should thus not lead to an exaggerated inflammatory response (8).

Since the experiments above are all performed *in vitro* on isolated cells, there is a possibility that a phenotype shift may occur during the short period of the assay and thereby skewing results. We therefore used our intravital imaging model (14, 15) to investigate efferocytosis and phagocytosis *in vivo*. Following intravenous injection of bacterial particles or neutrophils we could identify macrophages in the pancreas that had engulfed red-fluorescent material. Comparing exocrine-resident macrophages to endocrine-resident macrophages, we found similar differences in efferocytosis and phagocytosis compared to the *in vitro* results. We conclude from this that despite the lack of MMR expression in endocrine-resident macrophages, there does not seem to be any impairment in functional phagocytosis by these cells. Instead, an increased level of phagocytosis may influence the phenotype of a macrophage (9), and might thus be one of the cues from the microenvironment skewing endocrine-resident macrophages into an inflammatory subset. Since the endocrine part of the pancreas is more densely vascularized and receives 10-15% of the total pancreatic blood flow despite only consisting about 1-2% of its mass (30, 31), there is a possibility that deposition of *E.coli* bioparticles and dying neutrophils would be favored here. However, we used a significant time-period for (4 h post injection for bacterial particles and 24 h for neutrophils post injection) that would likely allow for equilibration across tissues.

With this study we sought to investigate some homeostatic and immune-defensive properties of endocrine-resident macrophages in healthy mice. Some aspects in the phenotype of endocrine-resident macrophages led us to hypothesize an impaired clearance of endogenous debris and bacteria. However, we found that endocrine-resident macrophages were more efficient in efferocytosis and phagocytosis, both *in vitro* and *in vivo*, than exocrine-resident macrophages. This difference in abilities may be one of the factors polarizing endocrine-resident macrophages to an inflammatory state since phagocytosis has been found to imprint macrophage heterogeneity. It remains unclear if this difference has any implications in the development of islet dysfunction or autoimmunity.

## Data Availability Statement

The raw data supporting the conclusions of this article will be made available by the authors, without undue reservation.

## Ethics Statement

The use of human biobanked tissue in this study was approved by the Regional Ethics Board in Uppsala, Sweden. The patients/participants provided their written informed consent to participate in this study. The animal study was reviewed and approved by Uppsala Region Laboratory Animal Ethics Board.

## Author Contributions

KP performed phagocytosis and efferocytosis experiments, flow cytometry, bioinformatics, statistical analysis and wrote the paper. NW performed cell isolation and qPCR. KM performed immunofluorescence staining and imaging. MK performed qPCR. RL performed flow cytometry analyses. GC conceptualized the study, performed intravital imaging experiments, statistical analysis, provided overall supervision and wrote the paper. All authors contributed to the article and approved the submitted version.

## Funding

Funding for this paper was provided by the Swedish Research Council, the Swedish Society for Medical Research, the Göran Gustafsson Foundation, the Lars Hierta Foundation, and the Family Ernfors Foundation.

## Conflict of Interest

The authors declare that the research was conducted in the absence of any commercial or financial relationships that could be construed as a potential conflict of interest.
