# Supplementary material for: Phagocytosis and Efferocytosis by Resident Macrophages in the Mouse Pancreas
Source: Front Endocrinol (Lausanne). 2021 May 25;12:606175. doi: 10.3389/fendo.2021.606175 (PMC8185276; doi:10.3389/fendo.2021.606175)
Supplement: Supplementary file 1 [file DataSheet_1.pdf]

SUPPLEMENTAL INFORMATION

Phagocytosis and efferocytosis by resident macrophages in the mouse pancreas

Kristel Parv, Nestori Westerlund, Kevin Merchant, Milad Komijani, Robin S. Lindsay, and Gustaf Christoffersson

Supplemental Figures 1-2

Supplemental Tables 1 and 2

Supplemental Figure 1

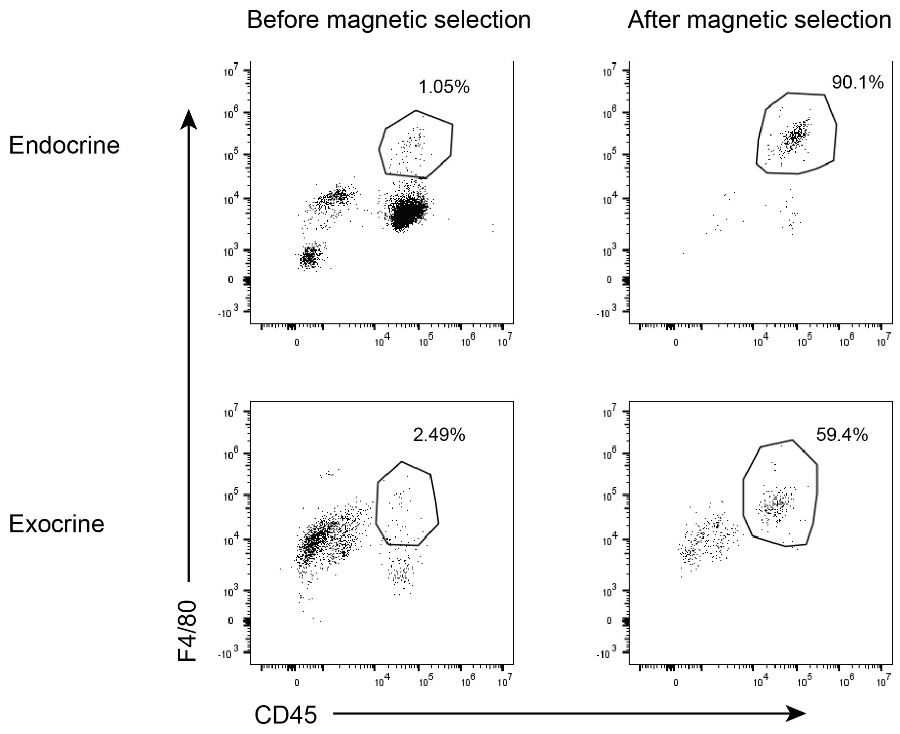

Efficiency of magnetic bead separation of macrophages from mouse pancreas. Endocrine and exocrine tissues were separated using collagenase digestion and density gradient centrifugation. Tissues were further processed to single cell suspensions, incubated with anti-F4/80 beads, and passed twice through magnetic columns. Representative flow cytometry plots with gates for macrophages are shown in the panels above. Debris and dead cells (Aqua live/dead marker) were excluded from the analysis.

Supplemental Figure 2

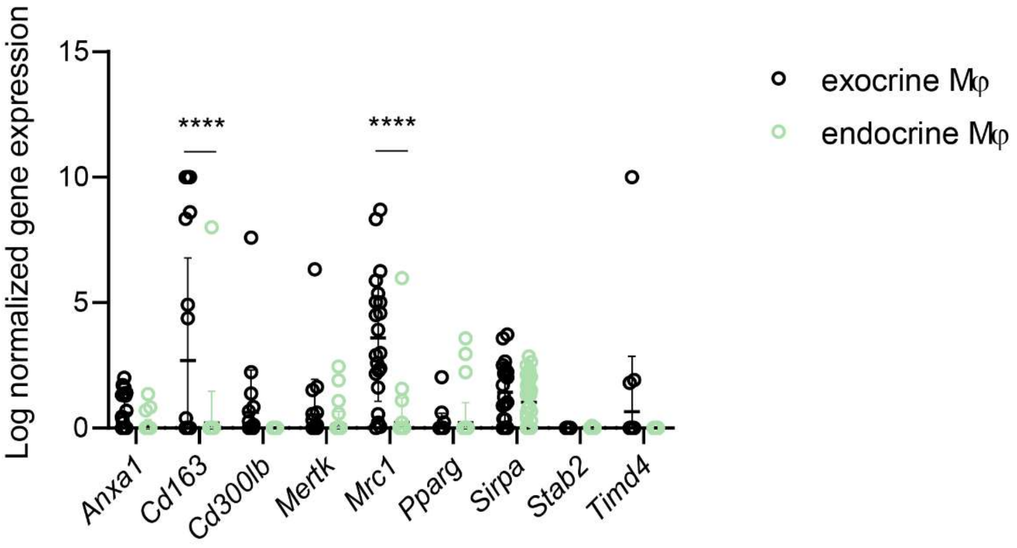

Validation of qPCR results to an open scRNAseq database. Data from the open database *Tabula Muris Senis* was used to validate qPCR data. A similar non-difference to the data produced in our own lab was found as only the expression levels of *Mrc1* and *Cd163* were significantly different.

Supplemental Table 1. Antibodies used in the experiments.

Antigen	*Antigen localization*	*Primary antibody*	*Secondary (if applicable)*	*Isotype control (if applicable)*
CD45	Extracellular	Biolegend 103130	NA	Biolegend 400629
F4/80	Extracellular	Biolegend 123120	NA	Biolegend 400525
CD11b	Extracellular	BD Biosciences 562287	NA	BD Biosciences 562308
CD11c	Extracellular	eBioscience 47-0114-82	NA	eBioscience 47-4888-80
MHCII	Extracellular	eBioscience 56-5321-82	NA	eBioscience 47-4031-80
Arginase-1	Intracellular	R&D Systems IC5868A	NA	R&D Systems IC016A
MMR	Extracellular	R&D Systems AF2535	Invitrogen A21432	R&D Systems AB108C
CD86	Extracellular	ThermoFisher 11-0862-82	NA	NA
CD19	Extracellular	e-Bioscience 25-0193-82	NA	Biolegend 400521
CD31	Extracellular	Biolegend 102416	NA	NA
Insulin (human)	Intracellular	Jackson	Abcam (ab150185)	NA
CD68 (human)	Extracellular	Abcam (ab955)	ThermoFisher (A21127)	NA
MMR (human)	Extracellular	Abcam (ab64693)	ThermoFisher (A31573)	NA

Supplemental Table 2. RT-qPCR primer sequences

	Primer sequence (5'-3')	
	Forward	Reverse
Actb	GCCCTGAGGCTCTTTTCCAG	TGCCACAGGATTCCATACCC
Anxa1	CTTTGCCAAGCCATCCTG	ACCACCTTTGATCTGTAGGGT
Cd163	TCTCAGTGCCTCTGCTGTCA	TCTTCCTTGACTCTGACCGC
Cd300b	CACCCATATGCAAGGCCCAGCATTGGTGAGG	GCGGCCGCTTAGTAGACGTTCACTTTAAC
Mertk	GAGGACTGCTTGGATGAACTGTA	GGAACCTAGCTGGGTGGA
Mrc1	GCAAATGGAGCCGTCTGTGC	CTCGTGGATCTCCGTGACAC
Pparg	GGAAGACCACTCGCATTCCTT	GTAATCAGCAACCATTGGGTCA
Sirpa	TCGAGTGATCAAGGGAGCAT	CCTGGACACTAGCATACTCTGAG
Stab2	ATTGCTCTGGCTGCCTACTC	GTTGGCTGGCTTCTCACATC
Timd4	ATTCTCCCATCCACTTCACAG	CTATCTTCAGTGTTGTCTGGC
